# The Biomechanics of Indirect Traumatic Optic Neuropathy Using a Computational Head Model With a Biofidelic Orbit

**DOI:** 10.3389/fneur.2020.00346

**Published:** 2020-04-28

**Authors:** Yang Li, Eric Singman, Timothy McCulley, Chengwei Wu, Nitin Daphalapurkar

**Affiliations:** ^1^State Key Laboratory of Structural Analysis for Industrial Equipment, Department of Engineering Mechanics, Dalian University of Technology, Dalian, China; ^2^Hopkins Extreme Materials Institute, Johns Hopkins University, Baltimore, MD, United States; ^3^Wilmer Eye Institute, Johns Hopkins Medicine, Baltimore, MD, United States; ^4^Department of Mechanical Engineering, Johns Hopkins University, Baltimore, MD, United States

**Keywords:** finite element methods, brain injury, vision loss, optic nerve, head trauma, concussion, biomechanics, optic neuropathy

## Abstract

Indirect traumatic optic neuropathy (ITON) is an injury to the optic nerve due to head trauma and usually results in partial or complete loss of vision. In order to advance a mechanistic understanding of the injury to the optic nerve, we developed a head model with a biofidelic orbit. Head impacts were simulated under controlled conditions of impactor velocity. The locations of impact were varied to include frontal, lateral, and posterior parts of the head. Impact studies were conducted using two types of impactors that differed in their rigidity relative to the skull. The simulated results from both the impactors suggest that forehead impacts are those to which the optic nerve is most vulnerable. The mode and location of optic nerve injury is significantly different between the impacting conditions. Simulated results using a relatively rigid impactor (metal cylinder) suggest optic nerve injury initiates at the location of the intracranial end of the optic canal and spreads to the regions of the optic nerve in the vicinity of the optic canal. In this case, the deformation of the skull at the optic canal, resulting in deformation of the optic nerve, was the primary mode of injury. On the other hand, simulated results using a relatively compliant impactor (soccer ball) suggest that primary mode of injury comes from the brain tugging upon the optic nerve (from where it is affixed to the intracranial end of the optic canal) during *coup countercoup* motion of the brain. This study represents the first published effort to employ a biofidelic simulation of the full length of the optic nerve in which the orbit is integrated within the whole head.

## Introduction

Indirect traumatic optic neuropathy (ITON) is a mechanical impact-induced injury to the optic nerve that often results in partial or complete loss of visual function to one or both the eyes (1, 2). It is distinct from direct traumatic optic neuropathy, a condition in which a fragment from a fracture of the skull or a penetrating foreign body directly damages the optic nerve. One estimate reports that ITON occurs in 0.5 to 5% cases of closed head trauma (3). ITON is an important medical problem associated with non-penetrating injury to the head because of the potential for devastating effects on vision. In this study, we perform mechanics-based simulations to quantify the likelihood and predict the location of ITON from impact forces to the head.

The incidence of ITON has not been directly reported in a large civilian population. However, surveillance studies of traumatic optic neuropathy of any type have been reported for pediatric and adult populations in England. For both adults and children, the overall incidence of either direct or indirect traumatic optic neuropathy is ~1/million (4, 5).

The mechanism of injury associated with ITON is poorly understood. Neither the location along the length of the optic nerve where the injury occurs nor the magnitude or type of forces that cause the injury are known. It has been suggested that there might be direct damage to the optic nerve axons and/or to the vascular supply of the optic nerve (6–8) and it is clear that there is ultimately retrograde damage to- and loss of retinal ganglion cell axons (9–11). Diffusion tensor imaging studies (DTI) have demonstrated reduced fractional anisotropy along the nerve (12) and ocular coherence tomography suggests that the retinal nerve fiber layer may acutely thicken, as well (13). Currently, there are no confirmed protocols for prevention, mitigation or treatment of ITON (2).

Two aspects of ITON that seem to be widely accepted are that it seems more likely to occur after frontal injury and that it can occur from a relatively minor impact insufficient to cause facial fractures (14). However, these observational findings have neither been fully confirmed nor explained. Because the vision loss associated with ITON can be devastating, understanding the biomechanics of this condition in humans is imperative to guiding preventive, mitigating, and therapeutic intervention.

We hypothesize two modes of optic nerve injury leading to the development of ITON: (a) Focused impingement of *stress* waves from the skull at the optic canal; and (b) Stretching deformation of the optic nerve between brain and the optic-canal due to *coup-countercoup* motion of the brain. We further hypothesize that impacts to the forehead are more susceptible to optic nerve injury compared to the lateral and dorsal impacts.

## Background

There are a number of suggested mechanisms by which blunt trauma leads to ITON (3), including (1) shear stress damage to the optic nerve sheath (along with its trabeculations and capillaries) and axons (14), (2) tugging of optic nerve between brain and the optic canal (15), and (3) nerve sheath hematoma and/or edema within the optic canal and chiasm (1). Clinical or cadaveric data that might validate one of more of these mechanisms is lacking. It has been proposed that the fusion of bone periosteum and optic nerve dura at the entrance, exit and within the optic canal make the optic nerve more susceptible to deformation of the optic canal and the relatively lower elastic tissues adjacent to it (10). Adding to this complexity is that ITON may be accompanied by damage to the orbit making it difficult to distinguish and enumerate the effects of direct and indirect energy to the nerve. Furthermore, the concussion associated with ITON may be accompanied by transient amnesia, making it difficult for patients to recount the event and provide information that might be valuable in understanding the mechanism of ITON. While CT and MRI are conventionally employed for diagnosing optic nerve and canal injuries (1), these modalities cannot detect ITON in the acute setting.

Finite element (FE) simulation with high-fidelity computational models based upon MRI data have been commonly regarded as a powerful mathematical method to evaluate the effects of traumatic injury. Uchio et al. (16) firstly developed an elaborate eyeball model and conducted the quasi-static uniaxial strip tests to measure the material properties and failure strains of the cornea and sclera. Stitzel et al. (17) considered the material of the eyeball computational model as non-linear elasticity and set up a validation protocol to predict globe rupture injury after comparing between their simulations and experiments. In addition, Cirovic et al. (18) included the globe, the orbital fat, the extra-ocular muscles and the optic nerve in their computational model to study the protective mechanism of eye stability during head trauma. The incompressibility of the orbital fat and the rigidity of the orbital walls can effectively restrict the excessive distortion of the eye and the optic nerve. Subsequently, several finite element models were developed with more accurate anatomy structures to fit different purposes such as the effects of impact, blast, and shaking, as well as eye mechanics and accommodation, respectively (19–22).

With the development of a traumatic brain injury (TBI) simulation, i.e., the reconstruction of an entire head model assembled with an orbital model, studies of indirect trauma on the globe have become possible (23–26). Huempfner-Hierl et al. (27) developed a simplified skull model to simulate the anterior impact on the forehead and showed that stressors propagated toward the optic foramen and the chiasm through the orbital ceiling. Notably, the majority of these computational models were employed to study globe rather than nerve injury. The etiology of ITON is still unclear due to the absence of a complete optic nerve FE model.

In this study, we developed a complete FE ITON computational model that includes the entirety of both optic nerves, globes and the supporting components, such as the orbital fat, extra-ocular muscles (EOM), as well as the brain, meninges and cerebrospinal fluid (CSF). We then used this model to explore the mechanics of ITON comparing a variety of injury sites and impact angles for a rigid and compliant impactor.

## Methods

### The Computational Head Model With High-Fidelity Orbit

The head model was built from MRI images of a 50th percentile male based upon the Visible Human Project supported by NIH. The Visible Human Project provides complete, anatomically detailed 3D human body images to the public. The MRI data used in our study consisted of axial slices of a male head and neck at 4 mm intervals. The MRI images were 256 by 256 pixel resolution with each pixel made up of 12 bits of gray tone (28). Materialize Mimics, a 3D medical image processor, was used to segment the head images to different components and convert the selected components to a surface model, which were then converted to volumes. These volumetric FE models were meshed in Altair Hypermesh software based upon the surface model. MRI (T1-weighted) images were segmented into different substructures, which included brain, ventricles, cerebrospinal fluid (CSF), subarachnoid structure (SAS), neck, and skull. Selected orbital substructures that were reproduced from MRI include fat, optic nerve, globe, bone, and extraocular muscles (EOMs).

Thinner structures such as the meninges were not resolved in the MRI images. Therefore, we manually created elements for the dura and pia by making a copy of the topology of surfaces in their neighborhood. Dura was created by shrinking the inner surface of the skull, and pia was created by dilating the outer surface of the brain (and the optic nerve). The skin and the cornea-sclera were developed from MRI images. Neighboring substructures that were in contact share common finite element node forming a perfect / no-slip interface, e.g., between fat and the orbit.

**Figure 1:**
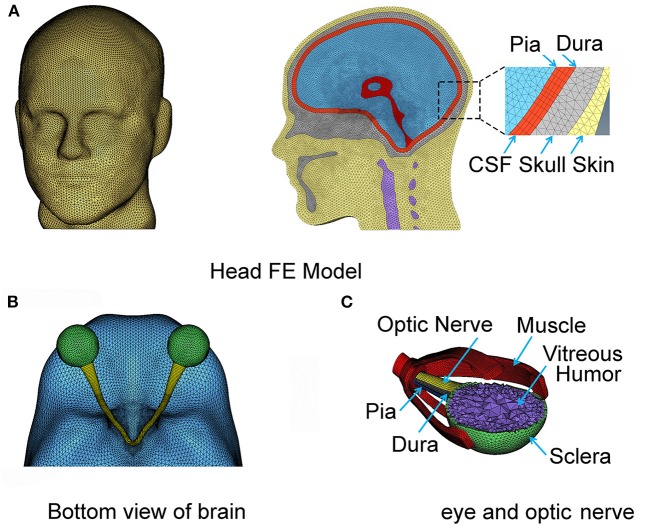
The complete FE head model with the meninges and CSF **(A)**. The eyeball and optic nerve are also included in the head model **(B,C)**, respectively.

As shown in Figure 1A, the skin model is meshed through to the skull and the cervical vertebrae. The CSF and the ventricles are modeled as soft solids, using a very low value of shear modulus that yields a corresponding Young's modulus of 1 kPa. This approximation allows us to consider the compliance of the fluid, circumventing the challenges associated with having to model the solid-fluid interface. The dura and the pia are assigned as membrane elements with thicknesses of 0.3 (29) and 0.13 mm (30), respectively. The arachnoid mater of the brain is not explicitly modeled; instead we homogenized the properties of sub-arachnoid space and CSF. Since CSF flows in the sub-arachnoid space, we assumed that the net compliance of the homogenized space can be approximated as a relatively soft solid (31). The CSF is commonly regarded as a soft solid in our model, with a bulk modulus of 2.1 GPa (32–34). The mechanical properties of these layers are poorly known, and thus most previous studies have made similar approximations when investigating the shear motions of the brain during mild accelerations.

The right and left hemispheres of the brain were separated by the falx cerebri as the mid-sagitally-oriented extension of the dura. The tentorium cerebelli is an axially-oriented extension of the dura that distinguishes the cerebrum and the cerebellum. The FE nodes of the falx and tentorium are numerically “tied” (i.e., no slip contact) to the finite elements of the substructure in the immediate vicinity. The biofidelic model of the brain sub-structures utilized in this work, including the meninges, was rigorously validated against *in situ* measurements of 3D brain deformations under mild angular accelerations of the head under sagittal and axial rotations (33, 35). It is recognized that the orientation of axonal fiber bundles within the white matter of the optic nerve is important for estimating the axial strains along the fiber.

The geometry of the eye and the optic nerve with its surrounding orbital tissues was also digitally reconstructed using Hypermesh software (by Altair Hyperworks), guided by the MRI images, as shown in Figure 1B. Since this study focuses on the optic nerve, the globe is simplified in our model with only a sclera and cornea layer enveloping vitreous humor. The EOMs are attached to the sclera anteriorly and extend posteriorly to the annulus of Zinn (a ring of fibrous tissue surrounding the optic nerve at the anterior opening of the optic canal). The extracranial optic nerve is connected to the posterior sclera of the globe and extends to the intraorbital opening of the optic canal where the nerve sheath attaches and remains attached until the nerve exits at the intracranial opening of the canal. The nerve then continues until it forms with optic chiasm by connecting with the optic nerve from the fellow eye as shown in Figure 1C. Notably, the length of the nerve in the orbit is longer than the distance from the eye to the globe, allowing the nerve to be slack within the orbit; the intracranial portion of the nerve is not slack. The nodes on the surface of the optic chiasm are numerically “tied” (no-slip contact) to the finite elements of the brain.

Normally, the dura and pia mater extend into the optic canal and envelop the optic nerve. We assumed that the SAS between the dura and the pia had a relatively lesser role in load transfer of the optic nerve, because of its smaller thickness compared to the SAS of the brain. For that reason, the SAS between the dura and pia of the optic nerve were excluded in the model.

All six EOMs muscles were included within the model for the orbit. These muscles are important for the motion of the globe within the orbit during normal functions. In addition, these muscles offer stiffness to the connection between the globe and the skull. The four rectus muscles (superior, lateral, medial, and inferior) are modeled using a solid finite element. Thicknesses of these muscles were explicitly modeled by referencing MRI images. The rectus muscles connect the globe to the posterior orbit. The two oblique muscles (superior and inferior) were relatively difficult to identify from the MRI images because of the constrained space in which these are located within the orbit. We employed a simplified structural form of the oblique muscles using a shell form of finite element with a finite thickness (5 mm, from MRI) to implicitly model the stiffness of their connection between the globe and the skull. The superior oblique muscle runs from the globe through a pulley-like trochlear notch in the superonasal orbit to the posterior orbit. The inferior oblique runs under the globe from its lateral aspect to an attachment in the inferonasal orbit. There are 12 main substructures to our computational head model as listed in Table 1, and each of these substructures is numerically tied with the its neighboring components by sharing the common nodes at the interface.

**Table 1:** Material properties of the computational head model.

	**Element type and number**		**Density(kg/m^**3**^)**	**Material properties**	**References**
Skull	Solid, C3D10M	1,056,814	1,300	E = 14.5 GPa, ν = 0.35;	(36)
Brain	Solid, C3D10M	3,262,394	1,040	E_0_ = 3.1 kPa, ν~0.5; *g*_1_ = 0.450, τ_1_ = 0.5; *g*_2_ = 0.365, τ_2_ = 50	(37)
Skin	Solid, C3D10M	311,390	1,200	E = 1 MPa, ν = 0.45;	(38)
Dura	Membrane, M3D4R; thick 0.3 mm	52,303	1,130	E = 31.5 MPa, ν~0.5;	(29)
Pia	Membrane, M3D4R; thick 0.13 mm	77,107	1,130	E = 10.8 MPa, ν~0.5;	(30)
Ventricles, SAS and CSF	Fluidic Solid, C3D8R	1,019,914	1,000	K = 2.1GPa, G_0_ = 500Pa, G_∞_ = 0 Pa	(39)
Optic nerve	Solid, C3D10M	33,782	1,040	E_∞_= 30 kPa, ν~0.5; *g*_1_ = 0.450, τ_1_ = 0.5; *g*_2_ = 0.365, τ_2_ = 50	(40)
Cornea-Sclera layer	Solid, C3D8R	26,856	1,400	K = 3.571 GPa; ν~0.5	(41, 42)
Vitreous humor	Solid, C3D10M	152,104	1,006	K = 2.272 GPa; ν~0.5	(24)
Rectus muscles	Solid, C3D10M	51,604	1,200	E = 11 MPa, ν = 0.4;	(43)
Oblique muscles	Shell, S3R; thick 5 mm	1,044	1,200	E = 11 MPa, ν = 0.4;	(43)
Orbital fat	Solid, C3D10M	846,714	1,000	E_∞_= 1.5 kPa, ν~0.5; *g*_1_ = 0.9, τ_1_ = 0.5; *g*_2_ = 0.5, τ_2_ = 50	(44)
Steel Impact	Solid, C3D10M	85,040	8350	E = 100 GPa, ν = 0.37	(45, 46)
Soccer Ball	Shell, S3R; thick 5 mm	3,516	553	E = 100 MPa, ν = 0.45, m = 0.45 kg, P_inner_ = 0.11 MPa	(47)

Supplementary Figure 1 shows biofidelic construction of cervical vertebral structure for the neck, developed from the MRI. Vertebrae, from C1 to a partial structure of C5, are modeled as a fused bone structure in our computational model. Since we did not include the entire spine in our model, the soft tissue surrounds the vertebral structure.

The entire computational head was meshed using 6,840,422 finite elements, and a finite element convergence test was performed on the mesh size for the optic nerve to numerically obtain mesh-independent results on impact loading. The finite element mesh was imported in a non-linear finite element analysis package (Abaqus software by Simulia). Because we assumed that the whole head is symmetric about the sagittal plane, we developed a half model on one side of the sagittal plane and then mirrored the other side. The coordinate system is referenced as Anterior-Posterior (AP axis), Right-Left (RL axis), and Superior-Inferior (SI axis).

### Material Property

A complete list of material properties is listed in Table 1. All substructures were modeled using isotropic and homogenous material properties. An elastic material requires two independent constants: the Poisson's ratio (ϑ) and the Young's modulus (*E*) values; these were assigned based upon measurements reported in the literature. Soft substructures, such as brain, were modeled using a value of Poisson's ratio that is approximately 0.5 (e.g., 0.49999975 in Abaqus FE software). This is a relatively high value, signifying incompressibility of brain, fat, vitreous humor, and the ventricles, as opposed to more compressible structures such as skin and the muscles. The resulting value of the bulk modulus for individual substructures is calculated using *E* = 3*K*(1−2ϑ) and is presented in Table 1. The bulk modulus is an average value for the brain and is consistent with values adopted in previous studies (33).

Viscoelastic material properties were assigned to relevant substructures (brain, fat, and optic nerve), since measurements were available in the literature from studies employing nano-indentation and other experimental techniques; these were commonly used in published simulation studies (36, 37, 44, 48, 49). We adopted a linear viscoelastic model to describe the biomechanical behavior of all the tissue components in the head model. For a linear viscoelastic model, the time-dependent shear modulus (relaxation modulus) is independent of the strain magnitude. Although non-linear viscoelastic model would be more appropriate for parts of the orbit that are anticipated to experience magnitudes of strain >20% (of the original length), material properties for a non-linear model are unavailable in the literature.

In linear viscoelasticity, the relaxation shear modulus *G*_*R*_(*t*) is determined by the dimensionless function, *g*_*R*_(*t*), expressed as a Prony series of *N* viscoelastic constants ${ḡ}_{i}^{P}$ and $\tau_{i}^{G}$. The dimensionless function is given by

(1) $$\begin{array}{r} g_{R}\left(t\right)=1-\overset{N}{\underset{i=1}{\sum }}{ḡ}_{i}^{P}\left(1-e^{-t/\tau_{i}^{G}}\right) \end{array}$$

where *g*_*R*_(*t*) = *G*_*R*_(*t*)/*G*_0_, and *G*_0_ is the instantaneous shear modulus. The long-term shear modulus $G_{\infty }=1-\overset{N}{\underset{i=1}{\sum }}{ḡ}_{i}^{P}$ can be obtained when time tends to a long-term value, *t* → ∞.

### Loading Setup for Realistic Impact Analysis

The impact analysis was performed using a dynamic module in Abaqus FE software, employing an explicit numerical time-stepping procedure. We referred to earlier investigations for a variety of impactors employed during studies of head trauma (20, 45, 46). It is commonly accepted that the mass and momentum (product of mass and velocity) of impactors play an important role in defining the energies of the impactor. However, studies employing the effect of different relative stiffnesses of the impactors have not been reported. Therefore, we modeled impactors with two different stiffness values, i.e., one with greater and the other lesser stiffness than the skull.

**Figure 2:**
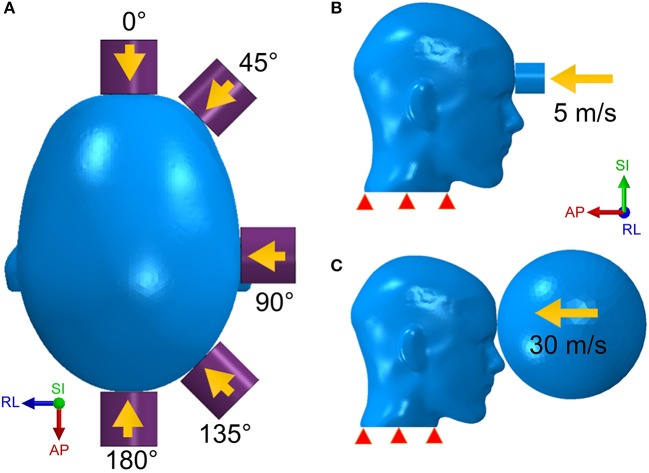
The head model is respectively struck by the cylinder impactor from five directions **(A)** with an initial speed of 5 m/s **(B)** and by the soccer ball with a speed of 30 m/s **(C)**.

We modeled the stiffer impactor as a cylinder with a density of 8,350 kg/m^3^, a Young's modulus of 100 GPa, a Poisson ratio of 0.37 and a finite element type as solid. Five impact regions as well as five impact angles were studied in the simulation (Figure 2A). The initial velocity of the impactor was set along the impact direction at 5 m/s (Figure 2B), which correlates with a general minor-impact type of blow such as from a fisticuff (50).

The less stiff impactor is made of a deformable elastomer sphere and was meant to simulate a soccer ball strike on the human head. The speed of a soccer ball can be as high as 25–35 m/s for case of high-powered shots taken by professional players (51). However, unlike the cylinder impact that strikes on a rather narrow area, the impact form of the soccer ball covers a wider area of the head due to the deformable nature of the soccer ball. In addition, the size of the soccer ball is comparable to the human head. For this case of loading, we built a hollow soccer ball FE model using a shell type finite element mesh with a diameter of 0.215 m, a mass of 0.45 kg and a fixed internal pressure of 110 kPa (51). As shown in Figure 2C, the soccer ball is placed in front of the forehead with an initial speed of 30 m/s.

## Results

### Cylinder Impactor

**Figure 3:**
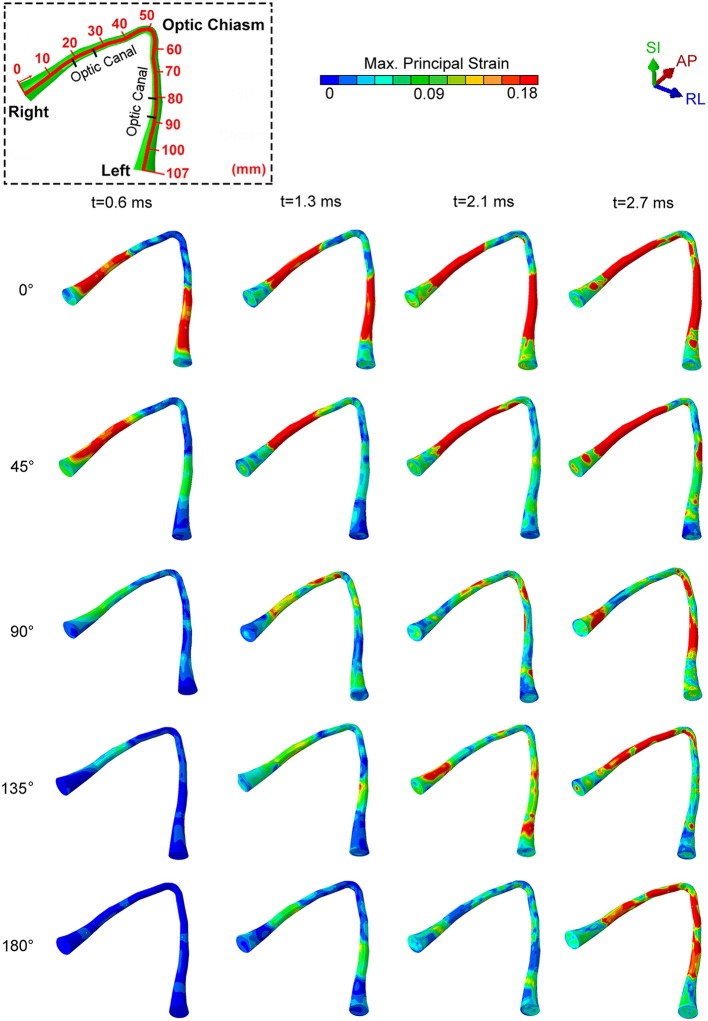
The locations of maximum principal strain of the optic nerve during cylinder impact for different impact directions.

Figure 3 shows snapshots of maximum principal strain distribution along the entire optic nerve under an impact of the cylinder at several different impactor orientations relative to the head. Bain and Meaney performed the tension experiments on the optic nerve of a guinea pig *in-situ* at strain rates of 30–60 s^−1^ to measure the functional and morphological tissue-level threshold strains for axonal injury due to stretch (52). In general, the functional threshold strains (corresponding to electrophysiological impairment) were less than morphological threshold strains (determined by immunohistochemical staining). Through regression analysis, liberal, conservative, and optimal strain thresholds of 28, 13, and 18%, respectively, were obtained for the onset of electrophysiological impairment. Based upon the previously successful application of the optimal strain threshold for injury analysis of the brain white matter (15, 34), we choose the same optimal strain threshold of 18% as a criterion for functional injury of the optic nerve. We assume a strong likelihood of functional injury if the strain at anytime during the dynamic analysis overshoots this strain threshold. We also assume that the likelihood of injury increases with an increasing extent of the optic nerve that is subjected to strain above this threshold. The dashed box on the top left part of the figure shows the position of the optic canal and optic chiasm relative to the optic nerve to aid in distinguishing those various regions of the optic nerve (i.e., intra-orbital, -canalicular, and -cranial).

For an impact direction (angle) of 0°, the optic nerve shows injury by 0.6 ms, and the injury sites initially occur within the optic canal, depicted by red colored regions in Figure 3. Subsequently, the likelihood of injury spreads rapidly to the remaining portions of the nerve, except the optic nerve head and the optic chiasm (Figure 3, t = 2.7 ms). The impact from 0° leads to a bilaterally symmetric injury of the optic nerves.

A 45° impact during which the impactor is brought to the right side of the forehead above the right eye resulted in a markedly asymmetric distribution of injury. In this case, there was injury only to the right optic nerve.

For the three other impact directions (i.e., 90°, 135° and 180° to the forehead), the optic nerve is subjected to strain below the injury threshold until 1.3 ms, but eventually shows some injured areas by 2.7 ms. Differences in the injury sites vary substantially for these three cases. At 90° and 135°, the impacts cause an asymmetrical strain distribution at 2.7 ms. The 90° impact leads to an injury near the optic nerve head for the optic nerve ipsilateral to the impact, while the injured regions are more distal to the optic nerve head for the optic nerve contralateral to impact. The 135° impact injures the ipsilateral nerve near or within the optic canal. In contrast, an impact at 180° causes symmetric injury to the optic nerves. However, unlike the 0° impact, the injured regions are more likely to lie in the posterior part of the optic canal, closer to the optic chiasm. In addition, the injury for the 180° impact develops much later in time (by almost 2 ms) compared to the 0° impact.

**Figure 4:**
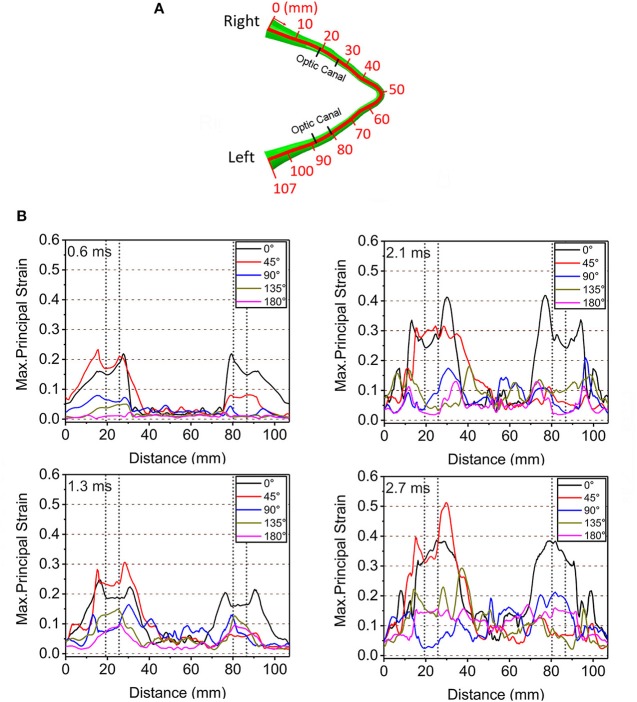
**(A)** Length scale along the optic nerve. **(B)** The maximum principal strain along the optic nerve at corresponding times of 0.6, 1.3, 2.1, and 2.7ms.

Figure 4 shows graphical representations of the average quantitative strain histories starting from the right optic nerve (secondary to a right-sided impacts angled at 45, 90° and 135°) and ending on the left optic nerve; this spans a length of approximately 100 mm, as shown in Figure 4A. The average is taken over four paths around the circumference (top, bottom, inside, outside) to demonstrate the distribution of strain over the length of the optic nerve, shown in Supplementary Figure 2. The strain value in the optic nerve increases with time. Impacts at both 0° and 45° cause higher strain value than the three other directions of impact. Paths corresponding to the 0° and 45° impacts shows elevated levels of strain in the vicinity of the optic canal, while the optic chiasm (at ~50 mm from the origin of the right optic nerve) is subjected to lower magnitudes of strain. Figure 4 also shows that there is symmetric pattern for strain associated with the 0° impact and an asymmetric pattern for strain associated with the 45° impact. In addition, the patters persist for the greatest duration compared to the other angles of impact. The greatest strain is seen for the 45° impact at 2.7 ms on the intracranial optic nerve ipsilateral to the impact, suggesting this is the most deleterious situation in terms of causing ITON, while the 0° impact is the next most severe case.

**Figure 5:**
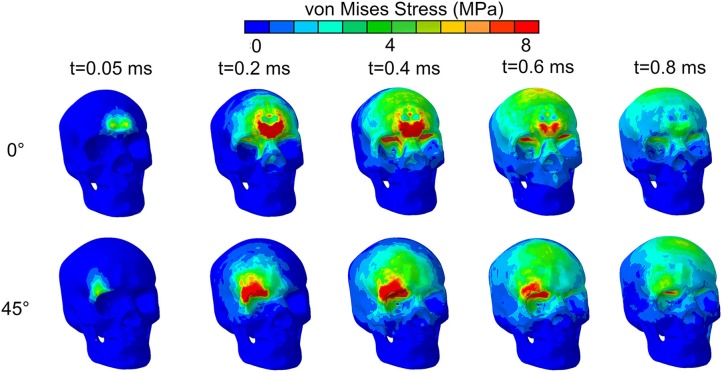
von Mises stress distribution on the skull for impact directions of 0° and 45° by an impact from a relatively stiff cylinder.

Further understanding on the role of stress waves in deforming the optic nerve between the 45° and 0° impact was obtained by analyzing the stress distribution in the skull from the moment of impact. Figure 5 shows the evolution of stress distribution over time on the skull for the 0° and 45 impact. Colors indicate magnitude of von Mises stress, a scalar measure obtained from the three-dimensional nature of the stress tensor (9 components in a 3 × 3 matrix). For both cases, the figure shows that the stress travels along the orbital ceiling and reaches the optic canal. For the 0° impact, a similar magnitude of stress reaches both orbits. On the other hand, for the 45° impact more stress reaches to orbit ipsilateral to the side of impact.

**Figure 6:**
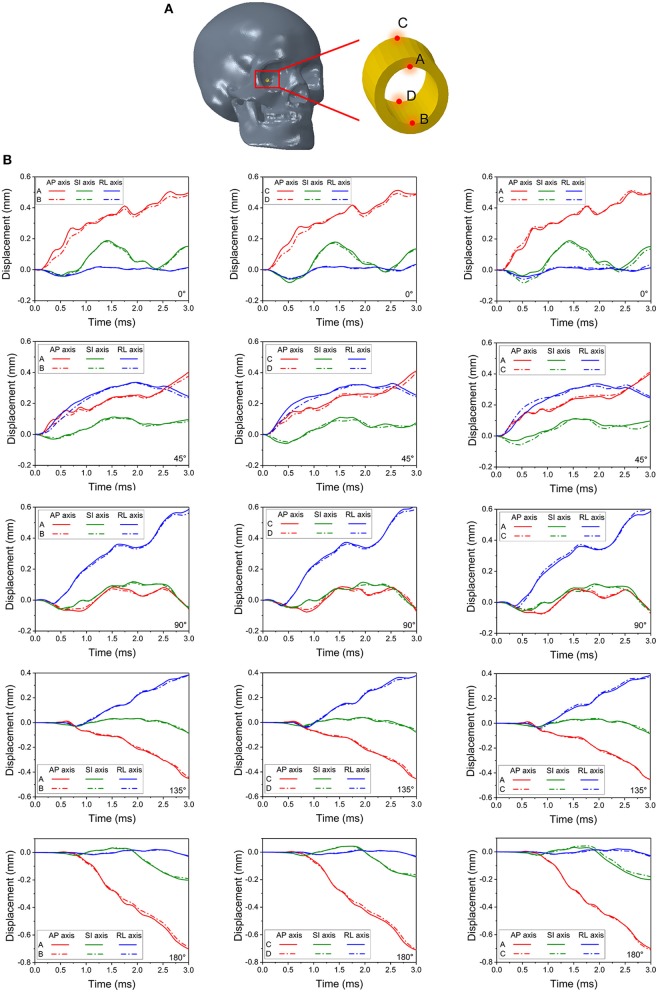
Four monitor points shown in **(A)** are set on the rim of the right (struck-side for 45°, 90° and 135°) optic canal indicate deformations of the canal in **(B)**.

Figure 6A depicts the potential locations of the bone deformations that might play a role in compression or shearing deformations of the optic nerve or optic canal. Displacements were measured at four locations: the intra-orbital (i.e., anterior) rim (point A for upper rim, point B for lower rim) and the intra-cranial (i.e., posterior) rim (point C for upper rim, point D for lower rim) of the canal ipsilateral to the impact. Figure 6B graphically portrays the differences in the displacements (i.e., relative motion) between respective upper and lower points, indicating the degree of deformation of the orbit. It should be noted that the relative displacement between points A and B along the SI axis will indicate a tendency to induce radially oriented compression/decompression deformations of the optic nerve, while displacement along the AP axis and RL axis will indicate a tendency to induce shearing deformations. When there is no relative displacement, then the displacement graph lines for the respective upper and the lower points will overlap. Further, differences between displacements of points A and C or between points B and D will indicate a tendency to induce either compression/decompression or bending of the optic nerve along AP-axis and RL-/SI-axes, respectively.

During the 0° impact, relative displacement of points A and B (also between C and D) along the AP-axis is higher than that along the SI- and RL-axis, indicating a predominant shearing force applied to the optic nerve along its length (within plane AP-SI). The difference in displacements can be noticed between 0.25 to 1 ms and again between 2 and 3 ms. Within these time windows, minor differences in displacements between points A and C along the SI-axis are also apparent, indicating a tendency for the optic canal to undergo bending in plane AP-SI. The deformations and displacements of the points along the RL-axis are relatively insignificant. Similar trends are seen with the 180° impact, although, the deformations of points A and B as well as points C and D along the AP-axis are smaller compared to the 0° impact. In contrast, the deformations for the 45° impact along the RL-axis are larger than those from the 0° impact. Displacement is apparent between points A and C and also points B and D, along the RL- and SI-axes, as well as between A and B and also points C and D along the SI- and AP-axis. This indicates that all deformations modes of the optic canal are active, i.e., shearing, compression/decompression, and bending. This further suggests that the 45° impact is indeed more deleterious compared to the 0° impact. Deformations for the impact at 90°, 135° and 180° progressively lessen and are smaller than those of the 45° and the 0° impact cases.

**Figure 7:**
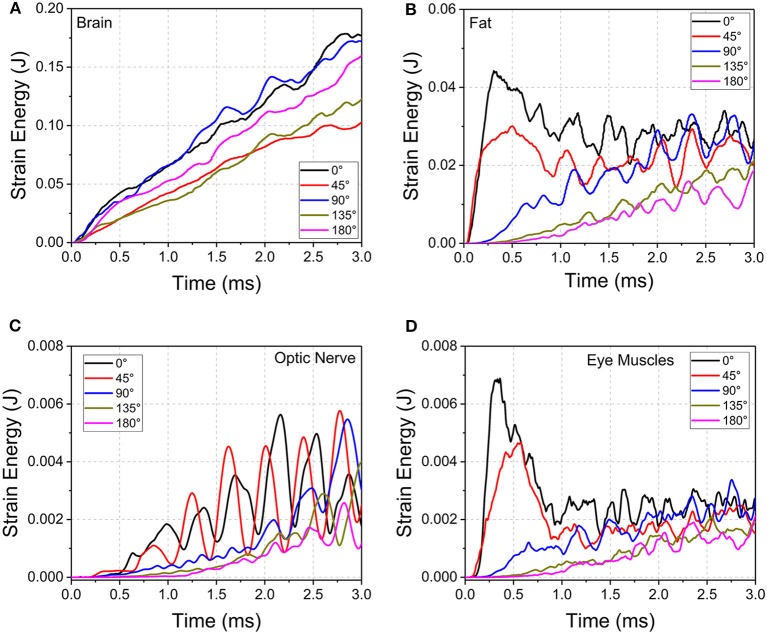
Strain energy development of **(A)** brain, **(B)** orbital fat, **(C)** optic nerve, and **(D)** eye muscles during cylinder impact for the different impact directions.

The evolution of strain energy (stored energy due to deformation) of the brain, orbital fat, optic nerve, and eye muscles were tracked for the entire duration of impact (Figure 7). The strain energy of the brain continuously increases for all the cases during the simulations. Notably for the 45° impact, the strain energy absorbed by the brain is the lowest compared to all other impact orientations, indicating an increased role of other substructures (besides the brain) in absorbing the impact strain energy. The 0° and 90° impacts led to the highest strain energies for the brain although the pattern of strain development is similar for all impact directions in that strain energy steadily rises over time.

For the orbital fat, optic nerve and eye muscles, the strain energies decrease as the angle of impact moves from 0° to 180° between 0 and 2 ms. However, the evolution of strain development differs among tissues and angle of impact. For fat and muscle, the strain energy peaks within 0.5 ms for 0° and 45° impact and then plateaus with oscillations while the strain energy for the other three directions steadily rises. For the optic nerve, the strain energy steadily grows from 0 to 3 ms for all angles of impact, although for 0° and 45° the strain energy demonstrates wide oscillations during this increase. The 45° impact led to the highest strain energy for the optic nerve, followed by the 0° impact.

Oscillations in strain energy development indicate the presence of stress waves traveling over the length of the optic nerve, which would induce oscillating deformations. The period of oscillations in the optic nerve are larger compared to the period of oscillations in the EOMs. This effect may be explained based on the characteristic material wave speed, defined as the square root of the ratio of material elastic modulus divided by material density. Deformation waves traveling back and forth over the length of the optic nerve result in the oscillations of deformation, and hence the oscillations in strain energy. Since the elastic modulus of the muscles is higher than that for the optic nerve by two orders of magnitude (Table 1), wave speed in the muscle tissue is higher compared to the wave speed in the optic nerve. Upon impact, the EOMs tend to absorb more strain energy, but this more quickly dissipates to substructures of the orbit because of the higher wave speed of the muscle. This would suggest that EOMs play a role in protecting the optic nerve during trauma.

### Soccer Ball Impact

**Figure 8:**
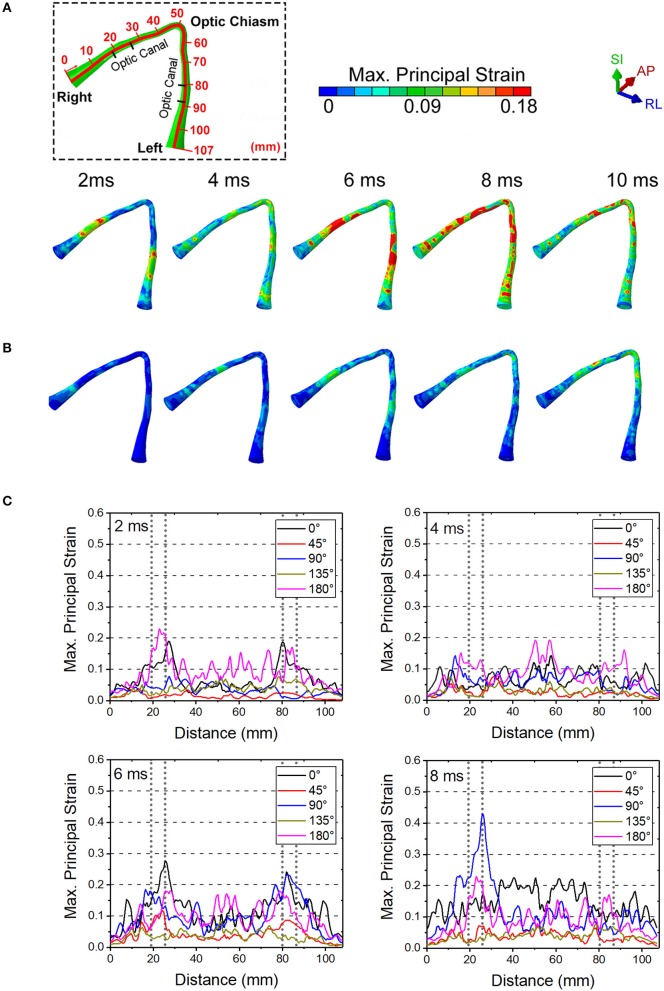
The maximum principal strain on optic nerve during the soccer impact from **(A)** 0 deg and **(B)** 45 deg. **(C)** Time history of strains for all five angles of soccer impact.

We performed simulations using the soccer ball for the same five impact angles explored with the cylinder. Figure 8 shows the maximum principal strain on the optic nerve during the soccer ball impact from 0° impact (Figure 8A) and 45° impact (Figure 8B). Comparison of these plots shows that the 0° impact demonstrates higher strains, indicating higher likelihood of injury compared to the 45° impact. Time histories of strain for all five angles of soccer ball impact are shown in Figure 8C. The 0° impact shows an oscillation of the strain forces; the magnitude of strain elevation is higher intraorbitally at 2 and 6 ms while it is higher intracanalicularly at 4 and 8 ms. The 180° shows a similar pattern of elevation at 2, 4, and 6 ms, but at 8 ms, the strain in the intraorbitally continues to rise. For both impact angles, there does not appear to be substantial differences between right and left nerves. The 90° impact shows higher strains later in time, indicating likelihood of unilateral injury after 6 ms. The 45° and the 135° impacts show the least magnitudes of strains among all the impact angles—this is in contrast with the case of cylinder impact.

**Figure 9:**
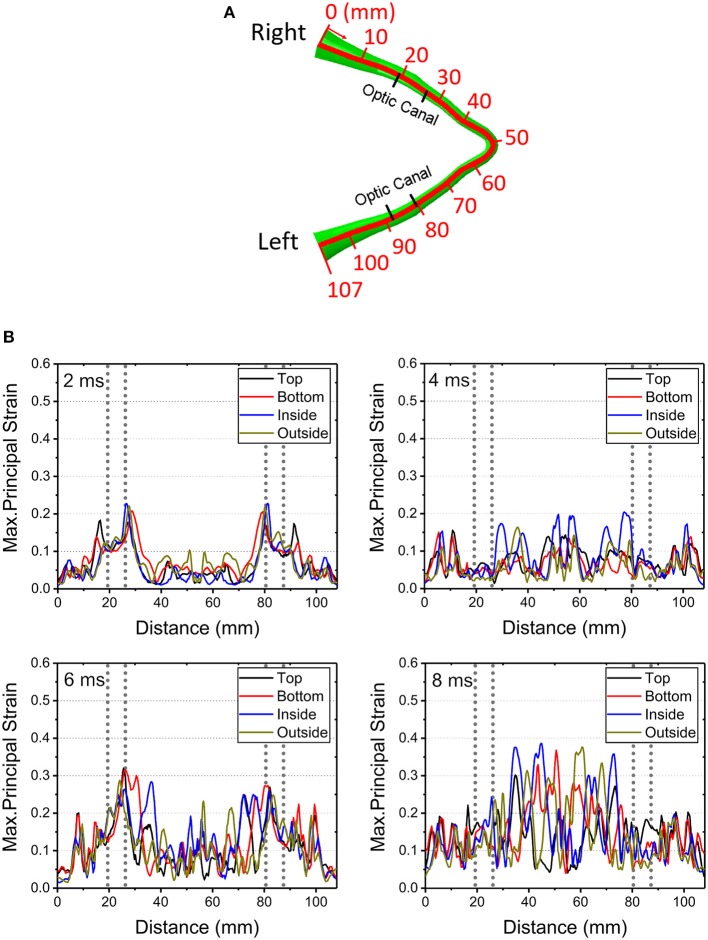
**(A)** Length scale along the optic nerve. **(B)** The maximum principal strain for four different paths along the length of the optic nerve optic nerve at times of 2, 4, 6, and 8 ms obtained from a 0° impact of a soccer ball.

Figure 9 shows the length scale along the optic nerve (Figure 9A) and the maximum principal strain for four different paths along the length of the optic nerve optic nerve at times of 2 ms, 4 ms, 6 ms and 8 ms obtained from a 0° impact of a soccer ball (Figure 9B). Supplementary Figure 2a shows chosen paths along four regions around the circumference (top, bottom, inside, outside) for distribution of strain along the length of the optic nerve. As with the cylinder impactor, it is presumed that the threshold for strain injury from soccer ball impact is 18% (34). These results show that strain at 2 ms is maximal at 30 mm and 90 mm, which are anatomically located within the intracranial region of the optic nerve. The result also shows that as time progresses the strain seems to increase near the chiasm so that by 8 ms it is greatest therein.

**Figure 10:**
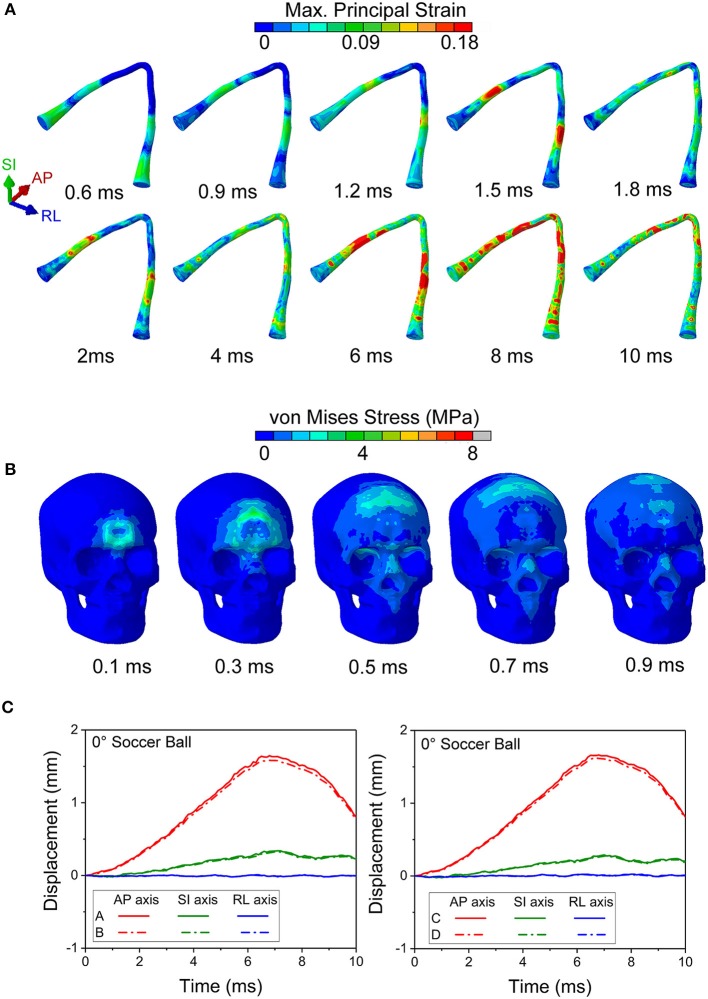
**(A)** Maximum principal strain of the optic nerve is presented during the 0° soccer ball impact. **(B)** Mises stress distribution of the skull during the 0° soccer ball impact. **(C)** Displacement of the optic canal during the 0° soccer ball impact.

Figure 10 shows maximal strain along the course of the optic nerve (Figure 10A), the von Mises Stress over the skull (Figure 10B) and the displacement for the points A-B and C-D (locations shown in Figure 6A) from the 0° impact angle with a deformable soccer ball (Figure 10C). Injurious levels of strain are first demonstrated by 4 ms from the time of impact. Injurious levels of strain are maximal at 6 ms and these energies are concentrated at the point of the optic where it exits the optic canal intracranially, then spreading posteriorly to include the optic chiasm.

Figure 10B shows that the stress wave propagates along the orbital ceiling, but the stress value is lower than that of the corresponding cylinder impact (Figure 5). Concerning potential deformation of the optic canal, no obvious displacement gaps can be found along SI- and RL- axes between the upper and lower rim (Figure 10C). There are some noticeable displacement differences between point A and B (also between points C and D) along the AP-axis, indicating a shearing mode of the optic nerve. These values indicate that the part of the optic nerve within the optic canal is indeed subjected to a finite strain, although the strain remains below the injury threshold for the most part of the impact duration, except at 6 ms (Figure 9B).

**Figure 11:**
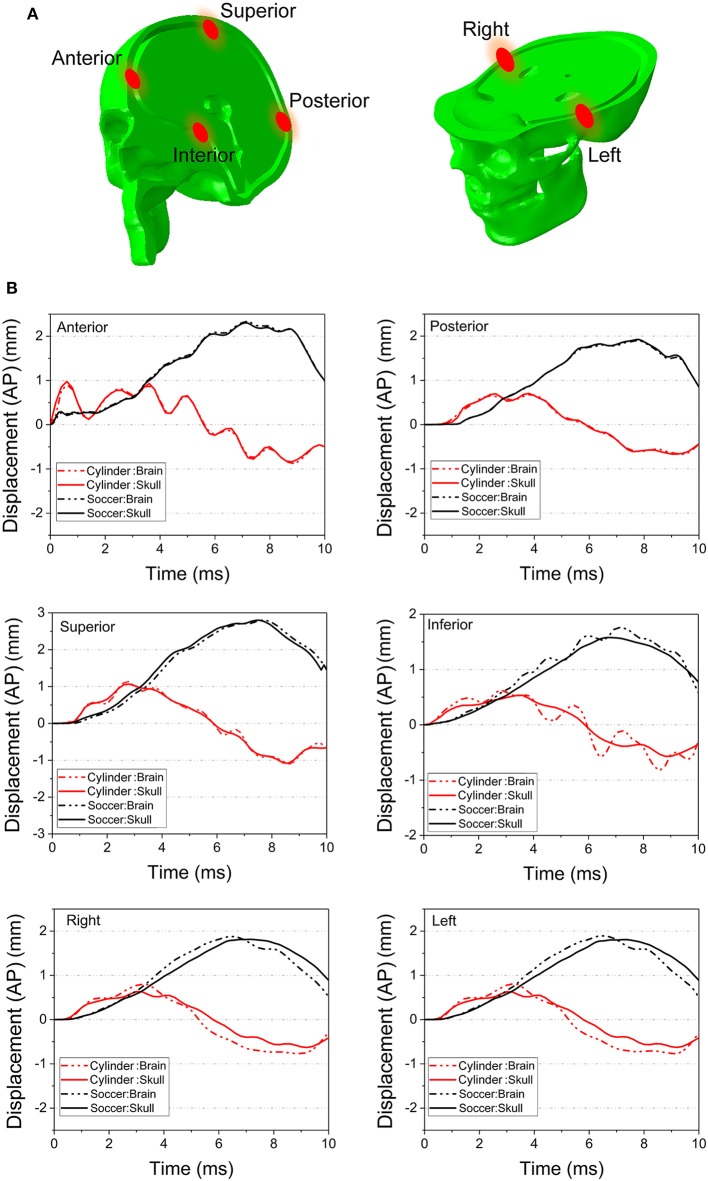
**(A)** Several pairs of monitor points are set nearby on the different area of the skull and the brain. **(B)** The displacement along AP axis of the monitor points are compared between the 0° cylinder impact and soccer ball impact.

Figure 11 compares the displacement of the head and brain along different axes for both the cylinder and soccer ball impactor at 0° impact. To clarify the role of brain motion in inducing injury to the optic nerve, the displacement along the AP axis for six (anterior, posterior, superior, inferior, right, and left) monitor locations are summarized and compared between the two types of impactors. Each location consists of a pair of points, one on the skull and another on the surface of the brain, as shown in Figure 11A. The difference between these corresponding points is essentially a measure of deformation of the sub-arachnoid space, i.e., the motion of the brain with respect to the skull. Figure 11B graphically depicts the motion of the brain-skull points for the two impactors between 0 and 10 ms. The absence of any gaps between the skull and brain graphs for the anterior, posterior and superior locations indicate negligible deformation. In the inferior region, the brain oscillates so that it is displaced more or less than skull, depending on the time. In the left and right locations, the displacement of the brain is initially greater than the skull until approximately 4 ms and then it is less than that of the skull. For the left, right and inferior locations, the differential displacement of the skull with respect to the brain could be expected to create forces that are transmitted to the intracranial optic nerve since the optic nerve is fixed at both the optic chiasm (to the brain) and intracranial optic canal (to the skull).

Concerning the overall movement of the head, in the cylinder case, the AP axis displacement indicates that the head initially moves posteriorly until ~ 3 ms and then rebounds, albeit with oscillations, particularly for anterior and inferior locations. On the other hand, the head motion in the soccer ball impact case completes only half a cycle movement, moving posteriorly until 8 ms. The maximal head movement is greater for the cylinder than for the soccer ball only until ~3 ms. Thereafter, the movement of the head continues to increase posteriorly for the soccer ball impact to a maximum of ~2 mm. Finally, it should be noted that the effect of relatively low shear modulus of the brain-skull interface compared to either brain or skull (Table 1) can be seen in Figure 11B. In particular, this figure shows the displacement difference at the brain-skull interface is highest at the inferior monitoring point, indicating that the inferior region of the brain-skull interface may be particularly prone to shear strain; it is in this location that the optic chiasm is located.

## Discussion

The simulations suggest that more than one possible injury mechanism might result in ITON. Notably, these results are in agreement with both static loading studies (6) and the blunt trauma simulations (27) that conclude that the forehead impact would lead to a stress wave that propagates through the orbital roof and concentrates at the orbital apex. However, the presence of an explicit model for an optic nerve in our case enabled us to perform advanced analyses of optic nerve injury site, pattern, and mechanisms of injury. In our simulations for the cylinder impact, the 0° and 45° cases demonstrated that the stress wave propagates from the superior orbital rim to the optic canal along the orbital roof (Figure 5). This stress wave resulted in an uneven deformation of the upper and lower rim of the optic canal that would cause a predominantly shearing type injury in the optic nerve. Notably, Santos-Bueso et al. (53) have reported a clinical case of ITON in which a patient suffered a right frontal impact, which is very similar with the 45° case in our simulation.

It also appears that the likelihood of injury to the intracanalicular optic nerve is higher than that for the intracranial and intraorbital portions of the nerve with the cylinder impact. The impact energy was propagated from the orbital ceiling to the upper rim of the optic canal, which leads to optic canal diameter reduction and damage to the optic nerve. Furthermore, the relatively higher strain energy of the orbital fat for the 0° and 45° impact cases indicates that the blunt impact around the orbital rim is more likely to cause injury at these angles than at 90°, 135° and 180°. Indeed, the corresponding deformations of the optic canal from cylinder impacts at 90°, 135° and 180° appear to be insufficient to cause injury within the optic canal (Figure 6). Moreover, the strain energy of the orbital fat is also extremely low at the beginning of the impact, supporting the idea that the deformation of the optic canal is not likely to cause any optic nerve injury in the 90°, 135° and 180° cases.

Concerning the less stiff impactor, we chose a soccer ball because TBI is unfortunately common in ball sports. The impact of the ball with the head can induce deformations of brain substructures beyond the functional injury thresholds (47). Indeed, TBI has been ranked as the most important cause of injuries in soccer (54, 55). Because of the deformable nature of the soccer ball, the skull stress is much lower compared to the 0° impact with the rigid cylinder. Although the initial impact area of the soccer ball is small (at 0.1 ms), this impact area keeps increasing since the ball deforms. The stress wave also propagates along the orbital roof, but the stress tends to dissipate before it arrives at the orbital apex. The symmetric displacement of the optic canal rim also suggests that there is no obvious deformation of the optic canal in case of the soccer ball impact. Rather, the inconsistent AP axis displacement of the skull and brain and the fact that levels of energy sufficient to damage the nerve injury are reached at approximately 7 ms suggest that ITON in this case is most likely caused by motion of the brain subjecting the intracranial optic nerve to tension. This idea is supported by the finding that large and often times oscillatory (i.e., *coup contrecoup)* motion of the brain develops after soccer ball impact (Figure 11B); such motion would cause the brain to pull on the intracranial optic nerve. This tugging damage could also be exacerbated by the continuous deformation of the brain resulting from the increasing strain energy of the brain with soccer ball impact.

We understand that there are limitations to this study. Even the best simulations require assumptions, particularly with employing those literature-derived metrics used to build the whole-head model. Furthermore, as mentioned previously, creating a nonlinear viscoelastic model for substructures of the orbit that experience magnitudes of strain > 20% (of the original length) could improve the accuracy of the model. It is also germane to mention that our anatomic assumptions made about the dura and pia reflect data reported for the brain but that these may be less accurate for the optic nerve itself.

It has been reported that ~60% of the extra-fascicular matrix of the human optic nerve is made of varying types of connective tissue (56). Therefore, the assumption that the human optic nerve has a Young's modulus of 30 kPa may be low. Published data of the cow optic nerve reports a Young's modulus of 5.2 MPa (57). This measurement by Shin et al. (57) on the intraorbital part of the cow optic nerve corresponds to the composite property of the optic nerve, including the pia. Results from our computational model using the higher end of the modulus, 5.2 MPa (57) are shown in Supplementary Figure 3. This simulated result indicates that the optic nerve does not lead to any injury to the intraorbital part of the optic nerve for the case of 45° impact at 50 m/s impact velocity, which is 10 times the injurious velocity (5 m/s) for the case with lower modulus (30 kPa). At such a high velocity, our simulations suggested the likelihood of skull fracture (judging from maximum principal stress exceeding fracture stress of the skull), while the injury of the optic nerve was precluded. Thus, a constant modulus of 5.2 MPa over the length of the optic nerve overestimates the stiffness of the optic nerve in our simulations.

On the other hand, *in vivo* shear wave elastography studies on normal humans indicated that the mean Young's modulus of the distal nerve was 17.3 kPa (58), which is comparable to our estimate of long-term viscoelastic modulus of 30 kPa. In our study, the composite modulus of the optic nerve, including the pia, is 0.67 MPa. Moreover, the modulus of the optic nerve is likely to vary over it's length, since the fraction of collagen in the optic nerve is known to decrease from intraorbital part to intracranial part (56). In addition, the amount of collagen also varies between animals and humans (56). With the lack of consistent and detailed measurements on the viscoelastic properties of the optic nerve, we use a constant value for the viscoelastic long-term modulus, 30 kPa, over the length of the optic nerve. Regardless of the actual value of the Young's modulus, the *relative* distribution of forces along the varying segments of the optic nerve measured in this simulation is likely to remain unchanged, as would be the conclusion that impacts at certain angles are more likely to cause ITON. The authors recognize that the current ITON head model still requires validation through animal (59) and cadaveric-based (60) experiments; this research is ongoing.

Future efforts with this model could be applied to other medical concerns that arise in patients with mild TBI. For example, our finding that the skull base substructures (such as pituitary gland) may be prone to injury could help clarify the biomechanics leading to neuroendocrine disorders reported after mild TBI (61–63).

In summary, we have created the first human whole head finite element model to include the orbits and the entire lengths of the optic nerves. This model has helped create data to suggest that impacts at the superior orbital rim (forehead) appear be those most likely to cause ITON. In addition, it indicates that there are two major injury mechanisms: (1) the uneven deformation of the optic canal, which further induces deformation of the optic nerve, and (2) the tugging between the brain and the optic nerve.

## Conclusion

For this study, a high-fidelity orbit model within a whole head model including the brain was developed and used to study the biomechanics of ITON. The cylinder impact on the ITON model from different directions have been simulated. The simulation results provide analysis of the maximum principal strain, von Mises stress, the strain energy, and the displacements within the optic canal and the head-brain interface, supporting the most likely cause for injury mechanism of the optic nerve.

The shear effect at the optic canal is the most likely cause of injury from forehead impacts (0° and 45°) received from stiffer objects, compared with other impact directions (e.g., back or side of the head). The impact stress wave propagates along the orbital ceiling to the optic apex. The onset of injury occurs at intracanalicular region of the optic nerve. More specifically, relatively higher strains are seen at the optic nerve where the nerve exits on either side of the canal, since the nerve is tethered by the canal. In the case of an impact to the forehead by a relatively compliant object (relative to the skull), the tugging (tension) of the optic nerve is the most likely mechanism of optic nerve injury. The motion of the brain within the skull is the cause of tensile mode of deformation of the optic nerve. This injury is most likely to occur in the intracranial region of the optic nerve. The *coup countercoup* mechanism of injury is dominant in this case of soccer ball impact and so one would expect the impact to the back of the head to have the same likelihood of causing injury as the forehead impact.

## Data Availability Statement

All datasets generated for this study are included in the article/Supplementary Material.

## Author Contributions

All authors listed have made a substantial, direct and intellectual contribution to the work, and approved it for publication.

## Conflict of Interest

The authors declare that the research was conducted in the absence of any commercial or financial relationships that could be construed as a potential conflict of interest.
