# Supplementary material for: The Biomechanics of Indirect Traumatic Optic Neuropathy Using a Computational Head Model With a Biofidelic Orbit
Source: Front Neurol. 2020 Apr 28;11:346. doi: 10.3389/fneur.2020.00346 (PMC7198902; doi:10.3389/fneur.2020.00346)
Supplement: Supplementary file 1 [file Data_Sheet_1.pdf]

Supplementary Material:

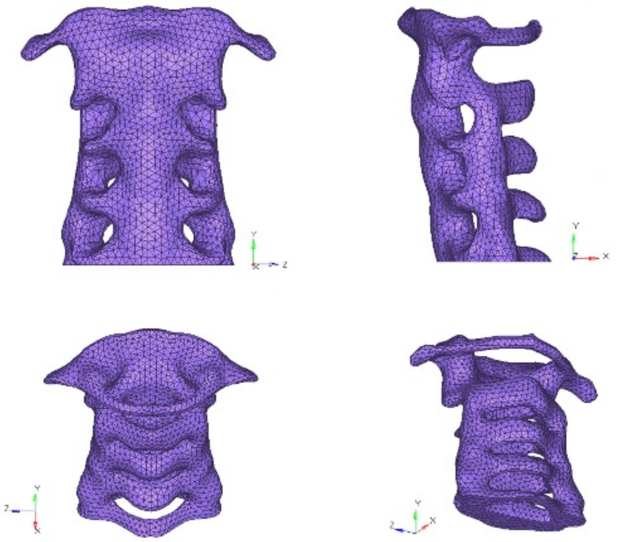

Supplementary Figure 1. Various views of the cervical vertebral structure for the neck. Vertebrae, from C1 to partial structure of C5, are modeled as a fused bone structure in our computational model. Since we did not include the entire spine in our model, the soft tissue surrounds the vertebral structure.

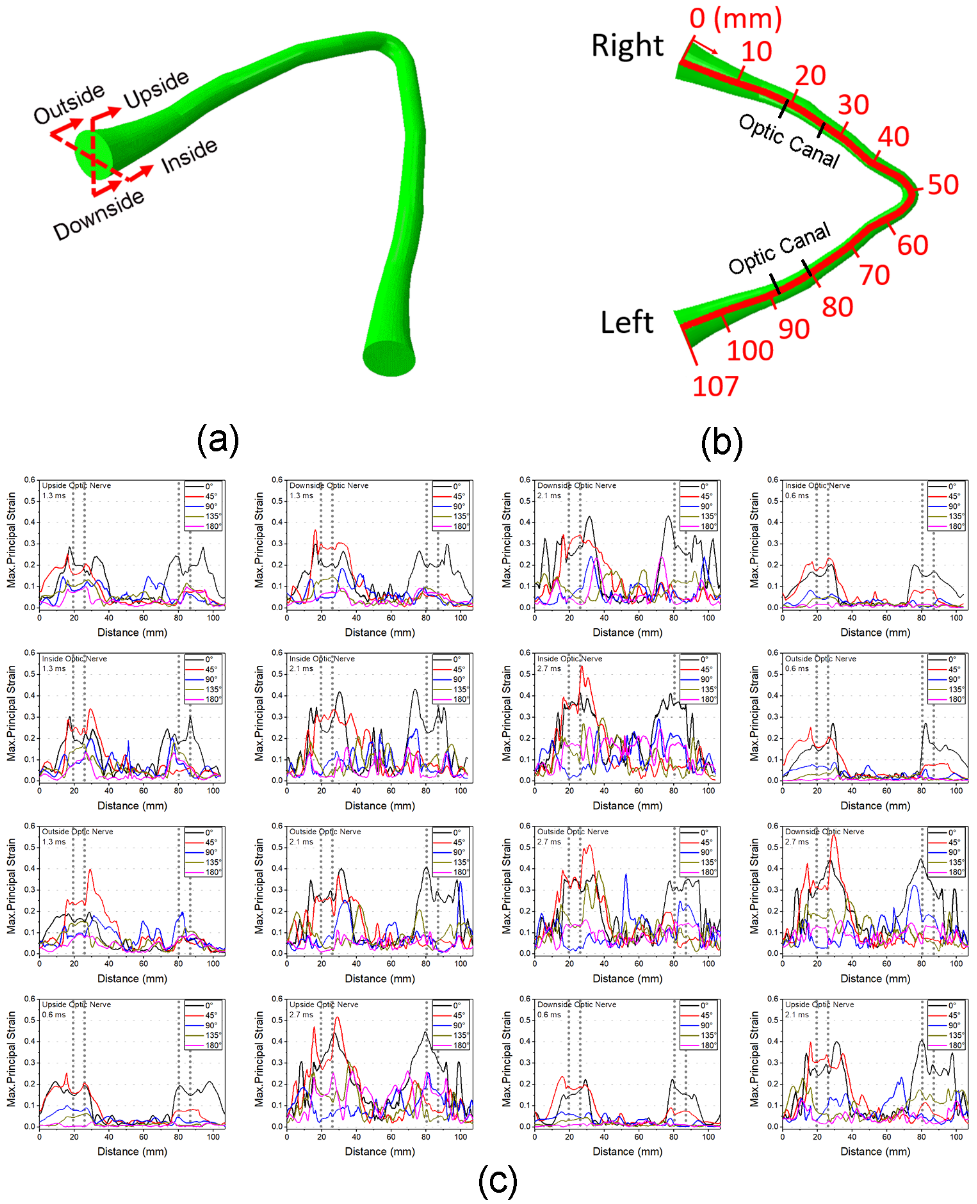

Supplementary Figure 2 The maximum principal strain of four different paths (a) length scale along the optic nerve, (b) on the optic nerve at corresponding times of 0.6ms, 1.3ms, 2.1ms and 2.7ms (c).

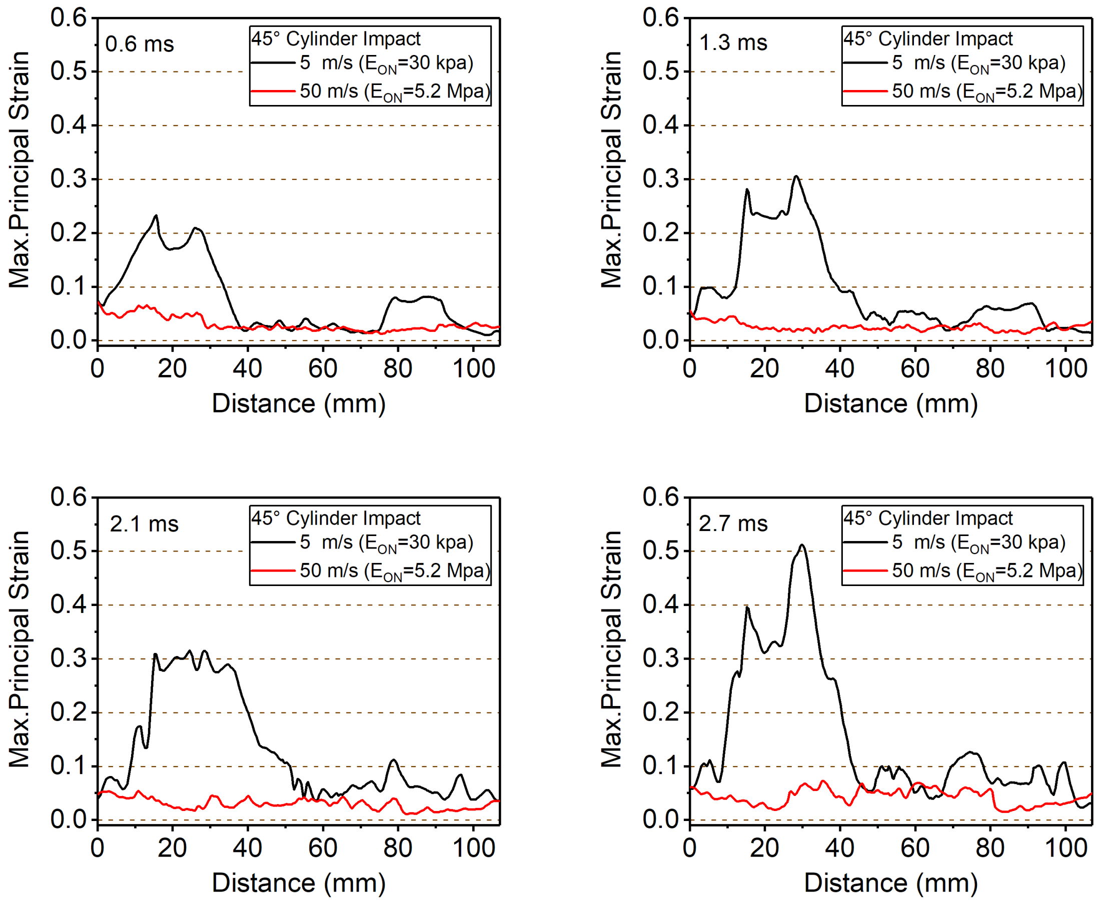

Supplementary Figure 3. Comparison of results for strain along the length of the optic nerve for two different long-term moduli of the optic nerve, 30 kPa and 5.2 MPa. The injurious strain (18%) was reached at 5 m/s impact velocity for the 30 kPa modulus, however, injurious strain was not reached for the 5.2 MPa modulus at 50 m/s impact velocity. Figures show the maximum principal strain along the optic nerve at corresponding times of 0.6 ms, 1.3 ms, 2.1 ms and 2.7 ms.
